# Numerical analysis of the slipstream development around a high-speed train in a double-track tunnel

**DOI:** 10.1371/journal.pone.0175044

**Published:** 2017-03-31

**Authors:** Min Fu, Peng Li, Xi-feng Liang

**Affiliations:** Key Laboratory of Rail Traffic Safety, Ministry of Education, School of Traffic & Transportation Engineering, Central South University, Changsha, Hunan, China; Beihang University, CHINA

## Abstract

Analysis of the slipstream development around the high-speed trains in tunnels would provide references for assessing the transient gust loads on trackside workers and trackside furniture in tunnels. This paper focuses on the computational analysis of the slipstream caused by high-speed trains passing through double-track tunnels with a cross-sectional area of 100 m^2^. Three-dimensional unsteady compressible Reynolds-averaged Navier-Stokes equations and a realizable k-ε turbulence model were used to describe the airflow characteristics around a high-speed train in the tunnel. The moving boundary problem was treated using the sliding mesh technology. Three cases were simulated in this paper, including two tunnel lengths and two different configurations of the train. The train speed in these three cases was 250 km/h. The accuracy of the numerical method was validated by the experimental data from full-scale tests, and reasonable consistency was obtained. The results show that the flow field around the high-speed trains can be divided into three distinct regions: the region in front of the train nose, the annular region and the wake region. The slipstream development along the two sides of train is not in balance and offsets to the narrow side in the double-track tunnels. Due to the piston effect, the slipstream has a larger peak value in the tunnel than in open air. The tunnel length, train length and length ratio affect the slipstream velocities; in particular, the velocities increase with longer trains. Moreover, the propagation of pressure waves also induces the slipstream fluctuations: substantial velocity fluctuations mainly occur in front of the train, and weaken with the decrease in amplitude of the pressure wave.

## Introduction

High-speed railways have undergone vigorous development in recent years. The geographical setting and requirements of the track regularity standard have resulted in a large number of tunnels. For example, there are over 300 tunnels in the Shanghai-Kunming High-speed Railway of China, with a total length of about 480 km. With increases in train speed, the instantaneous slipstream generated by moving trains may pose a safety threat to trackside workers and trackside infrastructure. When a train enters a tunnel at high speed, the air that originally occupies the tunnel space is pushed ahead and aside by the train nose, as well in open air. However, because of the confinement of tunnel walls, the airflow in a tunnel is significantly different than that in open air.

Transient slipstreams caused by high-speed trains traveling in open air have been widely and thoroughly investigated by the methods of full-scale tests, model tests and CFD simulations. The results include the following: the flow field in open air can be divided into several flow regions and the flow characteristics are different in each region [1–4]; the aerodynamic shape of the train and the transient slipstream can be related [5–6]; localized velocity peaks are found near the train nose and in the near-wake region [7–9]; The wake of high speed trains is a three-dimensional unsteady flow with a complex topology structure [10–12].

Researchers have also investigated the slipstream induced by high-speed trains passing through tunnels. A linear relationship between the slipstream velocity and the train speed in a tunnel has been discovered based on the data analysis of full-scale tests [13]. The air velocity and pressure distributions on the sides of high-speed trains in tunnels are investigated by utilizing the data of full-scale measurements [14]. The transient slipstream velocity gusts generated by high-speed trains in confined spaces have been researched by conducting a series of moving-model experiments at the Derby railway research center in the UK [15], and the physical nature of the wake flow in confined spaces has been examined by referring to velocity and static-pressure data from moving-model experiments and CFD simulations [16]. In contrast, with full-scale tests and model tests, numerical simulations can more easily supply comprehensive and systematic data describing the flow characteristics at a lower cost. One-dimensional models are often applied to predict the piston airflow inside tunnels based on the assumption that the induced flow caused by the piston effect in front of and behind the train is uniform in tunnels [17–20]. Simplified three-dimensional (3D) models in the study of the unsteady slipstream generated by high-speed trains in tunnels show that the airflow in tunnels exhibits 3D and highly unsteady characteristics [21–24]. The effect of tunnel length on the pressure transient and flow in single track tunnels were obtained by numerical simulations [25]. Although an investigation was recently conducted on the characteristics of the train slipstream in tunnels, elucidation of the detailed nature of the unsteady slipstream in tunnels requires further research.

The present study aims to use the 3D unsteady compressible Reynolds-averaged Navier–Stokes equations (RANS) and realizable k-ε turbulent model to investigate the characteristics of the slipstream caused by a passing high-speed train in double-track tunnels. The results from the simulation were compared with those from full-scale tests to verify the method used. The spatial slipstream velocity distributions around the train in tunnels and the time histories of the velocity components were investigated. The results of the piston effect on the features of the induced slipstream in tunnels were illustrated and compared to those in open air. Furthermore, the influence of different factors on the slipstream velocities, such as the train length, tunnel length, length ratio (train-length-to-tunnel-length ratio) and propagation of pressure waves in the tunnel, is discussed in this paper.

## Methodology

### Numerical method

When a high-speed train enters a tunnel, the surrounding flow field is highly unsteady, and the air in the tunnel cannot flow freely because of the confinement of the tunnel walls. The compressibility of the air subjected to the strong extrusion of the tunnel wall and car body should be considered. The FLUENT software was used to simulate the airflow in tunnels. Considering the computational cost, the 3D unsteady compressible Reynolds-averaged Navier–Stokes equations (RANS) were used, which have been proven to be efficient and robust in solving the flow field around a moving train [26, 27]. A realizable k–ε model is chosen to solve the turbulence flow in tunnels, because it is known to provide superior performance for flows involving rotation, boundary layers with adverse pressure gradients, separation and recirculation [28, 29]. The computational domain was discretized using the finite volume method (FVM). Simulations were performed using a pressure-based solver. The Navier–Stokes equations were solved using the second-order upwind scheme. The time derivative was discretized using the first-order implicit scheme for the unsteady-flow calculation in this paper. The SIMPLE algorithm was applied to solve the pressure-velocity field. The convergence criterion of the continuity equation was set as 10^−4^.

### Computational model and parameters

**Fig 1:**
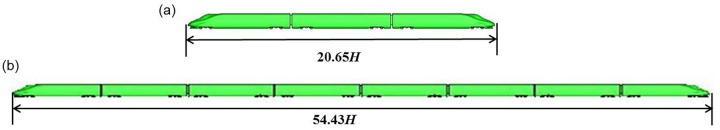
High-speed train models. (a) three-car grouping model; (b) eight-car grouping model.

Two simplified high-speed train-grouping models are used in this paper, the three-coach and eight-coach models, as shown in Fig 1. The geometric structures of the train body are smoothed with only bogies underneath and inter-carriage gaps, which are covered by semi-closed windshields. The height *H* of the train model is 3.7 m, which is considered as the characteristic dimension. The width of the train models is 0.91 *H*, and the total lengths of the two models are 20.65 *H* and 54.43 *H*, respectively.

**Fig 2:**
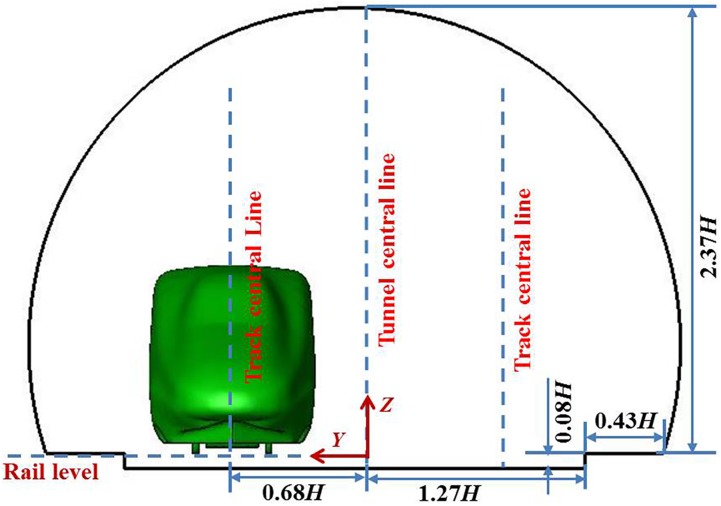
Contour of the tunnel cross-section.

The double-track tunnel is used for the 300–350 km/h high-speed railway in China with a cross-sectional area of 100 m^2^, and the track spacing is 5 m. The cross-section shape of the tunnel has a blockage ratio of 0.112, as shown in Fig 2, and the train runs on the left track with an offset from the centreline of the tunnel. Two tunnel lengths are considered in this paper: 135.13 *H* and 270.27 *H*.

**Fig 3:**
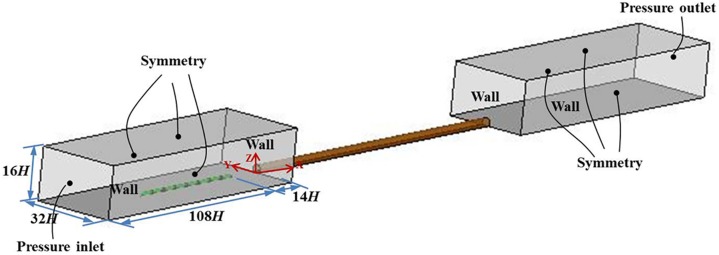
Computational domain and boundary conditions.

The computational domain with the coordinate system and boundary condition selections are illustrated in Fig 3. The outer zones in front of and behind the tunnel are 122 *H* long, 32 *H* wide and 16 *H* high. At the beginning of the simulation, the train nose is positioned at a distance of 14 *H* from the tunnel entrance. Relative to the running direction of the train, the origin point is shown in Fig 3, with the *X*-axis along the tunnel longitudinal, *Y*-axis along the tunnel lateral and *Z*-axis along the tunnel vertical direction. The train speed *V* is a constant. The dimensionless flow velocity components are *u*/*V*, *v*/*V* and *w*/*V*, and the dimensionless resultant velocity is defined as $U/V=\sqrt{u^{2}+v^{2}+w^{2}}/V$. The train-tunnel and train-ground relative movements are generated using the sliding-mesh technology [26, 30–31]. The moving boundary conditions applied to the train surface are as follows: the velocity component in the *X*-axis direction is equal to the train speed *V*, and both velocity components in the *Y* and *Z* directions are zero. No-slip walls are applied to the tunnel and ground. The inlet of the computational domain is set as the pressure inlet, whereas the outlet is set as the pressure outlet. The reference pressure in both the pressure inlet condition and pressure outlet condition is 0 Pa. The side and top boundaries of the outer zones are treated as symmetrical.

**Fig 4:**
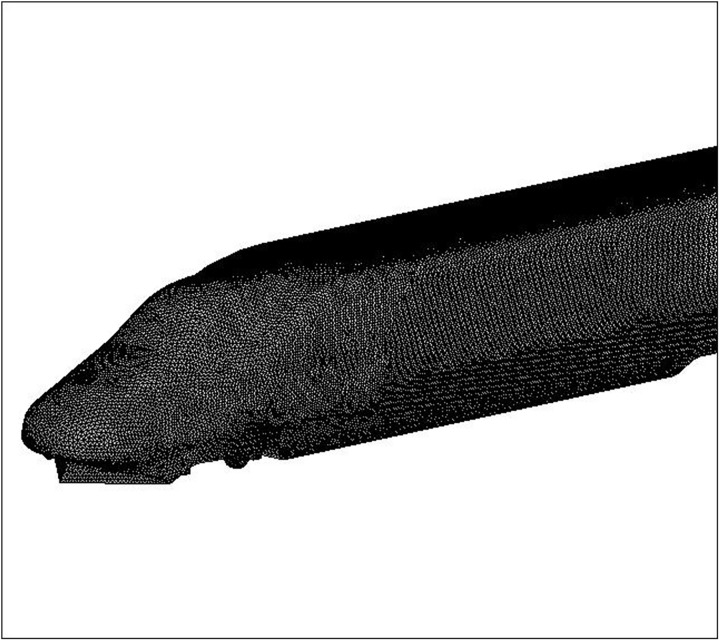
Surface grid of the train.

**Fig 5:**
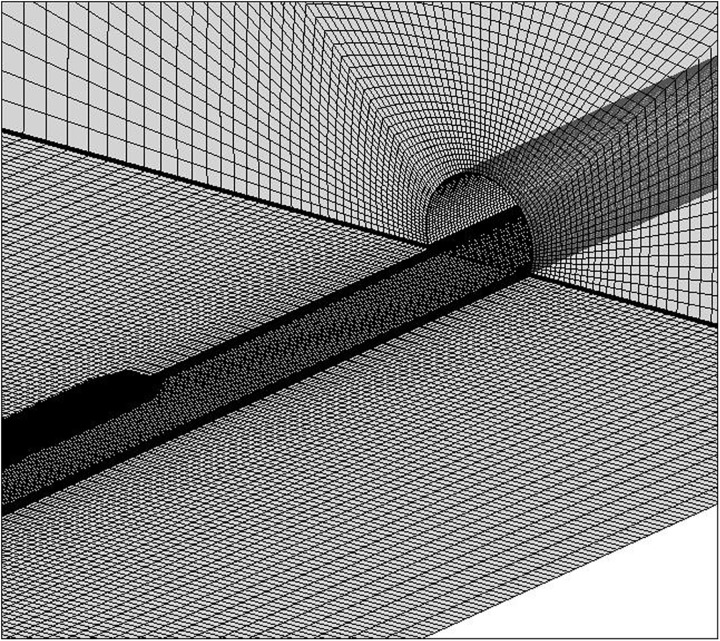
Grid around the tunnel entrance.

The computational domain is divided into several smaller zones and discretized by structured and unstructured hybrid grids. The body surface of the train is discretized by triangular grids, as shown in Fig 4. The volume around the train is discretized by tetrahedral grids, and other regions are discretized by hexahedral grids. Considering the effect of boundary layers on the surfaces of the tunnel and train body, the grids around the tunnel wall and train body are refined, whereas the grids in the outer zones far away from the train are relatively coarse. The smallest grid size around the train body and tunnel are 0.05 and 0.2 m, respectively. The transition region between refined mesh and coarse mesh is generated at a certain ratio. Fig 5 shows the computational grid around the tunnel’s entrance. To investigate the characteristics of slipstreams induced by high-speed trains in tunnels, three cases are simulated in this paper. The parameters of the tunnel and train and the total number of the grid for each numerical case are demonstrated in Table 1. In these simulations, the train is running on the left track with the train centreline at *Y* = 2.5 m, and there is no train on the other line. When the train nose reaches the tunnel entrance, the time is set as *t* = 0.

**Table 1:** Parameters for each calculation case.

Case	Tunnel length	Train length	Train speed	Grid cell numbers
**Case 1**	500 m	76.4 m	250 km/h	15 million
**Case 2**	500 m	201.4 m	250 km/h	16 million
**Case 3**	1 000 m	201.4 m	250 km/h	17 million

## Results and discussion

### Program validation

To validate the accuracy of the current numerical method, the simulation results of case 3 are selected to compare with the experimental data from the full-scale tests, which were performed on the Beijing-Shanghai high-speed railway of China in 2011. In the full-scale tests, the tunnel length is 1,005 m, the clearance area is 100 m^2^, and the line spacing is 5 m. The test train was the eight-coach CRH high-speed train, which passed through the tunnel at 250 km/h. An ultrasonic anemometer, used to measure the slipstream velocity, was placed 500 m from the tunnel inlet, 1 m from the train body and 1.5 m from the top of the rail (the height is essentially equal to the height of a standard adult’s gravity centre). The simulation conditions of case 3 are identical to those in the full-scale tests, and the time-history curve of the longitudinal airflow velocity is used to make a comparison.

**Fig 6:**
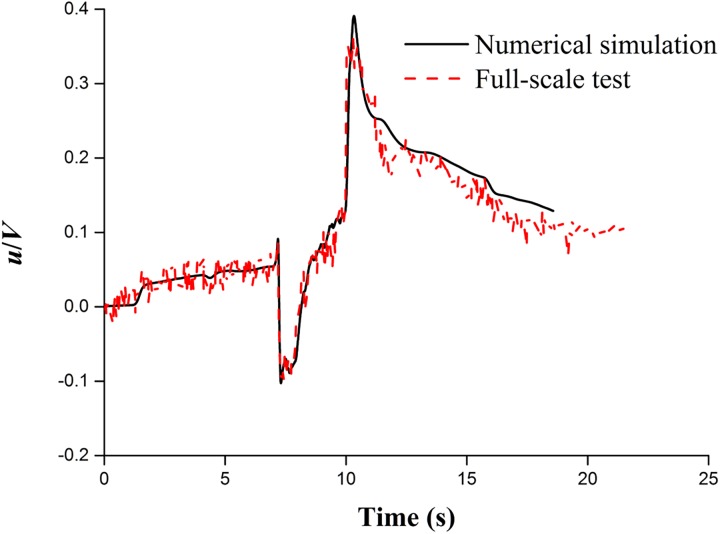
Comparison of the slipstream velocity from the numerical simulation and full-scale test.

Fig 6 shows the time histories of the longitudinal velocity component obtained from the numerical simulation and the full-scale test at the same measuring point. The simulation results are consistent with the full-scale data. However, the result curve from the simulation is smoother than that from the full-scale test. In addition, the maximum slipstream velocity is slightly higher than that from the full-scale test because the full-scale test is sensitive to the experimental field environment and measuring equipment. In general, compared with the full-scale data and simulation results, reasonable consistency is obtained, as shown in Fig 6.

### Spatial distributions of the slipstream velocity around the train in tunnels

**Fig 7:**
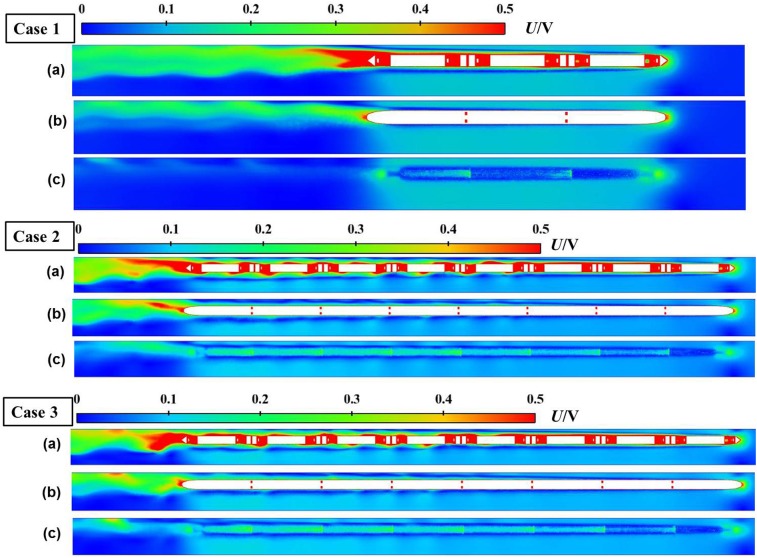
Velocity distribution around the train in the tunnel at different heights for cases 1–3. (a) *Z* = 0.25 m, height of the train bogie; (b) *Z* = 1.85 m, half of the train height; and (c) *Z* = 3.8 m, above the top of the train.

Fig 7 shows the velocity distributions on the horizontal sections of the tunnel with different heights at a certain time for cases 1–3. For cases 1 and 2, the train nose arrived at the middle of the tunnel (*X* = 250 m) at time *t* = 3.6 s; for case 3, the train nose arrived at the middle of the tunnel (*X* = 500 m) at time *t* = 7.2 s. The horizontal sections were located at *Z* = 0.25 m, *Z* = 1.85 m, and *Z* = 3.8 m, which are the height of the under-body structure, the half train height, and a point slightly higher than the top of the train, respectively. Fig 7 shows that the velocity distribution in the double-track tunnel is complex and that the effect of the spatial position is relatively large.

In the horizontal direction, the velocity distributions can be described as three distinct regions: the region in front of the train nose, the annular region between the train sides and the tunnel walls, and the wake region. The velocity distributions in front of the train are essentially symmetrical with the train centreline, whereas the velocity distributions in the annular region and wake regions are clearly non-symmetrical. For the annular region, the space on the left of the train is much narrower than that on the right side, which causes the movements of the airflows to become increasingly unbalanced between the left and right sides of the train along the train length. Thus, the slipstream velocities on the narrower side are more active, as shown in Fig 7. At the rear of the train, the attached boundary layer is separated from the tail head to generate numerous vortices, which subsequently shed into the near-wake flow and cause large fluctuations in the wake region. The unbalanced development of the boundary layer on both sides of the train causes the unbalanced development in the trailing vortex, which is clearly offset to the left-side space. Moreover, the diffusion of the trailing vortex is affected by the bounded airspace in the tunnel.

The velocity distributions in the vertical direction for all cases dramatically vary in height. The under-body complexities generate a complex flow in the under-body region, as shown in Fig 7(a). The largest slipstream velocity is obtained at *Z* = 0.25 m. The slipstream velocity decreases with the increase in height from the rail to the top. At a height of *Z* = 3.8 m, the speed variation of the flow around the train is relatively small because the active region of the tail vortex is mainly concentrated in the region below the half height of the train.

### Time-history analysis of the velocity components

**Fig 8:**
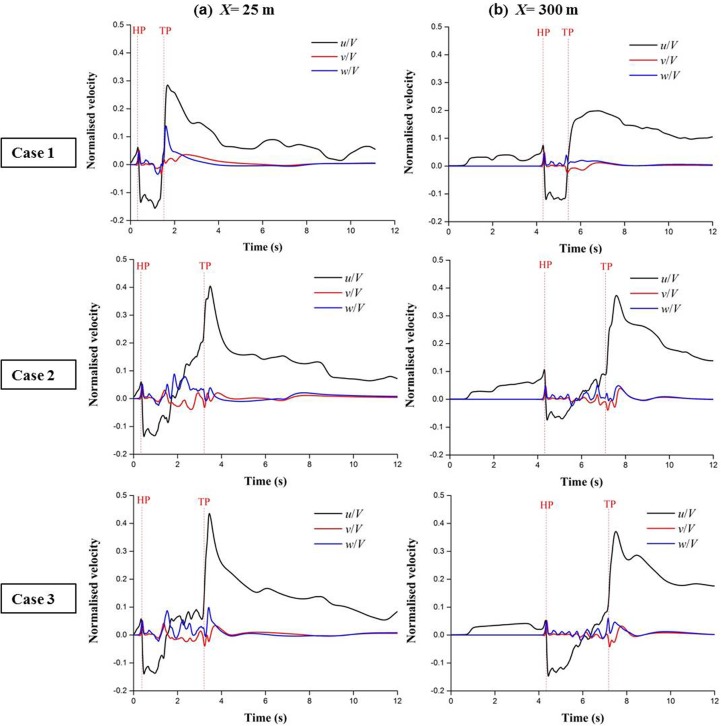
Time histories of the velocity components for cases 1–3 at two positions. (a) *X* = 25 m, *Y* = 5.5 m, and *Z* = 1.85 m; (b) *X* = 300 m, *Y* = 5.5 m, and *Z* = 1.85 m.

Fig 8 shows the time histories of the longitudinal (*u*/*V*), lateral *(v*/*V*) and vertical (*w*/*V*) velocity components at two monitoring points for cases 1–3. The times when the nose and tail of the train pass the monitoring positions are denoted by *t* = HP and *t* = TP, respectively. The flow patterns at different positions are similar in the three cases. The slipstream velocities are dominated by the longitudinal component, whereas the lateral and vertical velocity components do not appear to significantly affect the resultant velocity. The maximum velocity occurs in the near-wake region at the given positions for the three cases. Corresponding to the three regions mentioned in the previous section, the curves can also be divided into three segments with different characteristics according to the time of arrival or departure at the monitoring positions, as shown in Fig 8.

In segment I, there is only longitudinal airflow before the train nose reaches the monitoring positions in cases 1–3. The airflow speed slowly increases when the train approaches the monitoring point, but a few fluctuations occur in the curves. Before the train completely enters the tunnel, a longer length of the train entering the tunnel corresponds to a larger peak of the longitudinal velocity. Then, the longitudinal velocity gradually stabilizes after the entire train enters the tunnel. At *X* = 25 m, only one coach of the train has entered the tunnel when the train nose reaches the monitoring positions; thus, the velocity curves at this position are basically identical in cases 1–3. At *X* = 300 m, the speed variations in the three cases are different because of the effect of different train lengths and tunnel lengths, which will be discussed in the following Sections.

In segment II, all three components of the slipstream velocity frequently change. When the train nose arrives at the monitoring positions, the longitudinal velocities decreases, and a reverse flow is noticed in the longitudinal flow direction. The longitudinal airflow stalls at a negative value for a time until nearly three coaches passed the monitoring point, then it begins to gradually increase with the development of boundary layer regions of the train. When the train passes through the monitoring point, a part of the air is extruded by the train from the head towards the tail. The friction between the air and the walls of the tunnel and train causes transverse and vertical airflow. The lateral and vertical velocities are much smaller than the longitudinal velocity. Note that there are several apparent fluctuations on the curves of the three velocity components because of the effect of the inter-carriage gaps.

In segment III, when the tail car passes the monitoring positions, the surrounding air rapidly flows into the wake region because a large negative-pressure-dominated zone occurs behind the train. The values of the longitudinal components significantly increase to reach the maximum peaks and soon begin to decay. They are disturbed by the tail vortex and last for an extended time after the train leaves the tunnel. The lateral and vertical velocity components first increase in the near-wake zone and subsequently decay to zero.

### Piston effect

**Fig 9:**
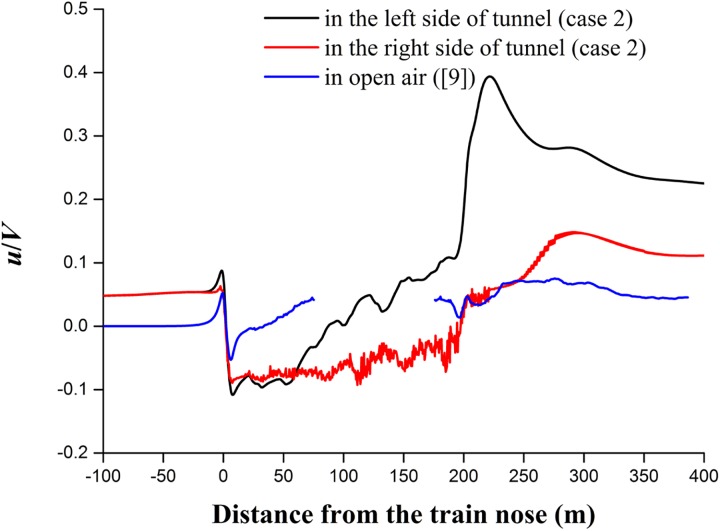
Comparison of the longitudinal velocity along the train for case 2 and open air.

Unlike the case in open air, a train that enters a tunnel acts as a piston that moves against the air that occupies the tunnel space, which is constrained by the tunnel walls. Thus, a “piston effect” is generated. To investigate the piston effect in a double-track tunnel, the slipstream velocities on two sides of the tunnel (e.g., case 2) were compared with those in open air [9]. Taking the trains as references, Fig 9 shows the comparison of the longitudinal velocity on two sides of the tunnel and in open air at a distance of 1.49 m from the train body and 2.05 m from the top of the rail. Because a 4-coach CRH2 train mode used in [9], a gap of the intermediate 4-coach length is observed in the open-air curve to make the comparison possible. On two sides of the tunnel, there are prolonged and more rapid airflows in front of and behind the train that moves in the direction of the tunnel exit compared to the case in open air. This feature is likely specific to the piston effect. A related discussion can be found in [15], which reveals that the piston effect appears to be a dominant cause for the increases in slipstream durations and peak magnitudes based on the result analysis of moving-model tests. In the tunnel, the airflow in front of the train nose is extended to the tunnel exit with the velocities remaining at approximately 0.09, whereas it is zero in open air. When the airflow reaches the nose tip of the train, the velocities generate the first peaks. The peaks at the two sides of the tunnel increase by approximately 197% and 138% compared to the velocities in open air. All of the largest slipstream velocities are obtained in the wake regions; the peak values in the tunnel reach approximately 470% and 246% compared to those in open air. The left side of the tunnel causes a greater increase than the right side in the double-track tunnels. These results suggest that the piston effect may be significantly affected by the space size between the train side and the tunnel wall.

### Effect of the train length, tunnel length and length ratio

**Fig 10:**
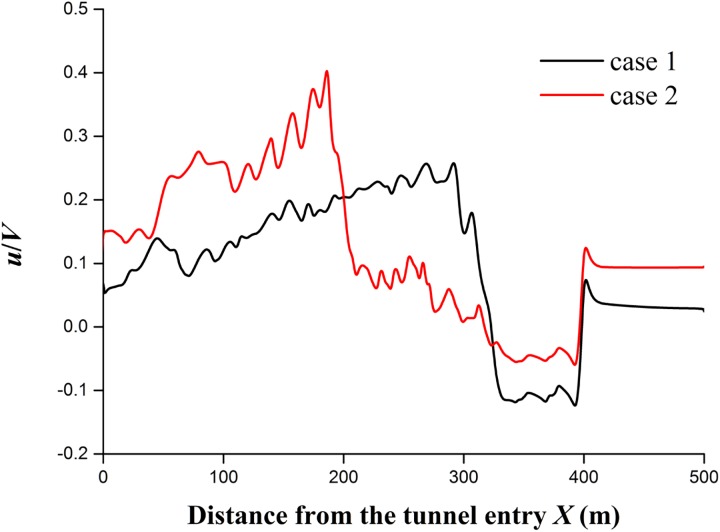
Longitudinal velocity distributions along the tunnel for cases 1 and 2.

**Fig 11:**
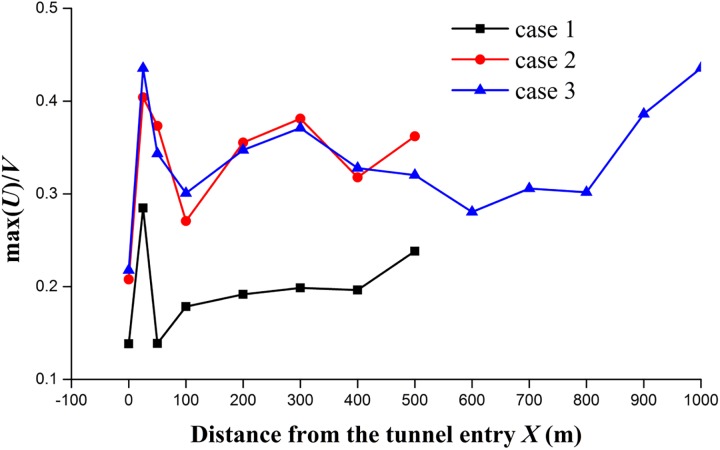
Peak values of the longitudinal velocities along the tunnel for cases 1–3.

To investigate the train length effect, two different grouping trains with the lengths of *L*_train_ = 76.4 m and 201.4 m are simulated in cases 1 and 2. The tunnel length is *L*_tunnel_ = 500 m in the two cases. The simulated results in cases 1 and 2 are shown in Fig 8. The change of train lengths causes a large change in the extreme values of the longitudinal airflow, which is along the train length. Fig 10 shows the longitudinal velocity distributions in the tunnel along a longitudinal line (*Y* = 5.5 m, *Z* = 2.05 m) when the train nose arrives at *X* = 400 m. The longitudinal velocity (*u*/*V*) ahead of the train nose in case 1 has almost more than doubled compared to case 2, possibly because the longer train in the tunnel causes a larger pushing force in front of the train and subsequently leads to a faster velocity of the air pushed in front of the train. The boundary layer grows along the train length, so different train lengths make the instantaneous flow structures in the annular and wake regions different between cases 1 and 2. The peak longitudinal velocity is approximately 0.28 in case 1 and 0.40 in case 2, as shown in Fig 11. The slipstream velocity is dominated by the longitudinal component, so the maximum slipstream velocity is increased by increasing the train length. On average, the peak values in case 1 are 56% higher than those in case 2. Therefore, to evaluate the safety of slipstreams in a tunnel, the effect of the actual train length should be considered.

**Fig 12:**
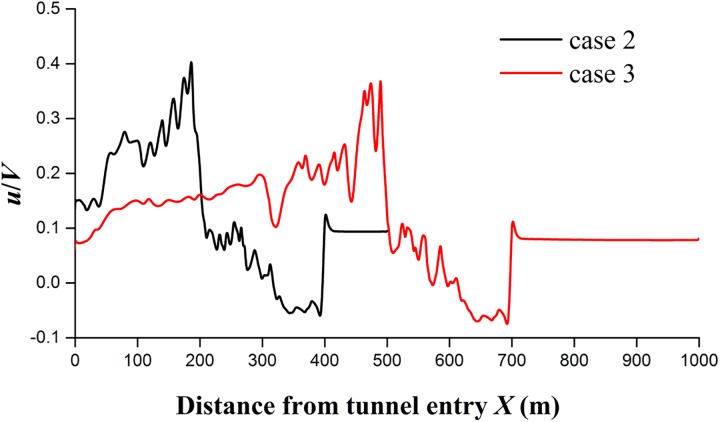
Longitudinal velocity distributions along the tunnels for cases 2 and 3.

The effect of the tunnel length on the slipstream velocity was studied by comparing the results of cases 2 and 3 with two different tunnel lengths: *L*_tunnel_ = 500 m and 1,000 m. The peak values vary according to the length of the tunnel. Clear differences between the two cases are observed at the entry and exit of the tunnels, whereas the differences are small at the positions inside the tunnels. In the tunnel with the length of *L*_tunnel_ = 1,000 m, there is a declining trend at *X*>500 m before the train begins to leave the tunnel. Fig 12 shows the longitudinal velocity distributions in the two tunnels along a longitudinal line (*Y* = 5.5 m, *Z* = 1.85 m) when the train nose arrives at *X* = 400 m and *X* = 700 m. These simulated results reveal that the maximum longitudinal velocity induced by the train in the tunnel slightly decreases when the train is farther along the tunnel. However, the effect of the tunnel length on the maximum longitudinal velocity is small relative to the train length.

Finally, the effect of the length ratio on the slipstream velocity was examined by comparing the results of the three cases as shown in Fig 11. The length ratio is defined as *L*_ratio_ = *L*_train_ / *L*_tunnel_. The length ratio is 0.1528, 0.4028, and 0.2014 in cases 1, 2, and 3, respectively. Although the maximum slipstream velocities at the entry and exit of the tunnels within a range of one train length are complicated and fluctuate in all three cases, the maximum slipstream velocities at the other positions are more stable with the decrease in length ratio.

### Effect of pressure changes

**Fig 13:**
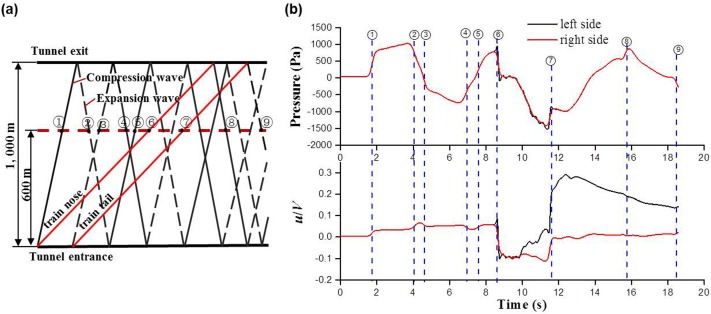
The static pressure and velocity variations in two sides of tunnel for case 2. (a) the wave diagram; (b) time history of the static pressure and longitudinal velocity.

When a train nose or tail enters/exits a tunnel, it generates compression or expansion waves which propagate at a nearly sonic speed and reflect from the tunnel entrance and exit. That causes complex pressure fluctuations inside the tunnel. Fig 13 shows the pressure and velocity variations of the entire time history at two symmetric points of Y = 6 m, Z = 1.85 m and Y = -6 m, Z = 1.85 m on the cross-section of *X* = 600 m in case 2. A wave diagram of the causes of pressure fluctuations in the time history is also included. The compression wave generated by the nose entering and leaving the tunnel is detected at (1) and (8), respectively. The expansion wave generated by the tail entering and leaving the tunnel is detected at (3) and (9), respectively. Reflecting pressure waves occur at (2), (4) and (5), and the train nose and tail pass at (6) and (7), respectively. The changes in static pressure and velocity occur almost simultaneously. When a compression wave arrives at *X* = 600 m, the static pressure significantly increases; the air is compressed and flows in the direction of wave propagation. When an expansion wave arrives at X = 600 m, the static pressure significantly decreases; the air is expanded and flows in the reverse direction of wave propagation. Thus, the airflow speed is increased or decreased, as shown in Fig 13(b). This effect is weakened with the decrease in amplitude of the pressure wave. The maximum amplitude of the velocity variations is 0.03 because of the first compression wave. Fig 13 also indicates that the static pressures at both sides of the double-track tunnel are almost identical, small differences of the static pressures only occur as the train passes through the measuring position. While the velocity differences occur as or after the train passes through the measuring position. In particular, the departure of the train tail generates a negative pressure zone behind the train and causes an abrupt increase in velocity at the left side of the tunnel but only slightly changes at the right side of the tunnel because of the greater distance from the train.

## Conclusions

Three cases were performed in this paper to investigate the slipstream velocity characteristics around high-speed trains in tunnels based on the 3D unsteady compressible Reynolds-averaged Navier-Stokes equations and realizable k-ε turbulence model. Numerical results were compared with the measurements from a full-scale test, and a good consistency was obtained. The following conclusions can be drawn.

1. The flow field around the high-speed train in double-track tunnels can be divided into three distinct regions: the region in front of the train nose, the annular region and the wake region. The slipstream velocity distributions show a significant spatial difference. The development of airflow is clearly offset to the left side space that is closer to the train. The higher airflow occurs in a lower-height region
2. The slipstream velocity is dominated by the longitudinal component, whereas the lateral and vertical velocity components do not appear to significantly affect the resultant velocity. The maximum velocity occurs in the near-wake region.
3. The piston effect causes prolonged and more rapid airflow in front of and behind the train that moves in the direction of the tunnel exit compared to those in open air. The slipstream velocity may be significantly affected by the space size between the train side and the tunnel walls.
4. The tunnel length, train length and length ratio affect the slipstream velocities; in particular, the effect increases with a longer train.
5. The propagation of pressure waves also causes the related slipstream fluctuations. The maximum amplitude of the velocity variations reaches 0.03 because of the first compression wave. Prominent velocity fluctuations mainly occur in front of the train and weaken with the decrease in amplitude of the pressure wave.
